# Effects of Six Weeks of Hypoxia Exposure on Hepatic Fatty Acid Metabolism in ApoE Knockout Mice Fed a High-Fat Diet

**DOI:** 10.3390/life12101535

**Published:** 2022-10-01

**Authors:** Yangwenjie Wang, Jessica Lavier, Weicheng Hua, Lijing Gong, Hao Wei, Jianxiong Wang, Maxime Pellegrin, Grégoire P. Millet, Ying Zhang

**Affiliations:** 1 Key Laboratory of Physical Fitness and Exercise, Ministry of Education, Beijing Sport University, Beijing 100084, China; 2 School of Sport Science, Beijing Sport University, Beijing 100084, China; 3 Institute of Sport Sciences, Faculty of Biology and Medicine, University of Lausanne, 1015 Lausanne, Switzerland; 4 Division of Angiology, Heart and Vessel Department, Lausanne University Hospital (CHUV), 1011 Lausanne, Switzerland; 5 China Institute of Sport and Health Science, Beijing Sport University, Beijing 100084, China; 6 Faculty of Health, Engineering, and Sciences, University of Southern Queensland, Toowoomba, QLD 4350, Australia

**Keywords:** hypoxia, hepatic lipid metabolism, diet, ApoE KO mice

## Abstract

Nonalcoholic fatty liver disease (NAFLD) is the most common liver disease with a characteristic of abnormal lipid metabolism. In the present study, we employed apolipoprotein E knockout (ApoE KO) mice to investigate the effects of hypoxia exposure on hepatic fatty acid metabolism and to test whether a high-fat diet (HFD) would suppress the beneficial effect caused by hypoxia treatment. ApoE KO mice were fed a HFD for 12 weeks, and then were forwarded into a six-week experiment with four groups: HFD + normoxia, normal diet (ND) + normoxia, HFD + hypoxia exposure (HE), and ND + HE. The C57BL/6J wild type (WT) mice were fed a ND for 18 weeks as the baseline control. The hypoxia exposure was performed in daytime with normobaric hypoxia (11.2% oxygen, 1 h per time, three times per week). Body weight, food and energy intake, plasma lipid profiles, hepatic lipid contents, plasma alanine aminotransferase (ALT) and aspartate aminotransferase (AST), and molecular/biochemical makers and regulators of the fatty acid synthesis and oxidation in the liver were measured at the end of interventions. Six weeks of hypoxia exposure decreased plasma triglycerides (TG), total cholesterol (TC), and low-density lipoprotein cholesterol (LDL-C) contents but did not change hepatic TG and non-esterified fatty acid (NEFA) levels in ApoE KO mice fed a HFD or ND. Furthermore, hypoxia exposure decreased the mRNA expression of *Fasn*, *Scd1*, and *Srebp-1c* significantly in the HFD + HE group compared with those in the HFD + normoxia group; after replacing a HFD with a ND, hypoxia treatment achieved more significant changes in the measured variables. In addition, the protein expression of HIF-1α was increased only in the ND + HE group but not in the HFD + HE group. Even though hypoxia exposure did not affect hepatic TG and NEFA levels, at the genetic level, the intervention had significant effects on hepatic metabolic indices of fatty acid synthesis, especially in the ND + HE group, while HFD suppressed the beneficial effect of hypoxia on hepatic lipid metabolism in male ApoE KO mice. The dietary intervention of shifting HFD to ND could be more effective in reducing hepatic lipid accumulation than hypoxia intervention.

## 1. Introduction

Nowadays, nonalcoholic fatty liver disease (NAFLD) is the most common liver disease worldwide [1,2] and includes a series of liver diseases, from simple hepatic steatosis (non-alcoholic fatty liver) to non-alcoholic steatohepatitis [3]; furthermore, non-alcoholic steatohepatitis may develop into cirrhosis and even liver cancer. Besides the liver-related morbidity or mortality, NAFLD also associates with a high risk of diabetes and cardiovascular diseases [4,5]. To successfully manage this condition, a growing number of studies are focusing on the pathogenesis of NAFLD to design more efficient therapeutic strategies [6].

Abnormal lipid metabolism is one of the major characteristics of NAFLD. Peroxisome proliferator-activated receptor α (PPARα) is expressed at high levels in the liver and is known to be involved in mitochondrial fatty acid β-oxidation [7,8]. The genes of rate-limiting enzymes in mitochondrial fatty acid oxidation, such as medium-chain acyl-coenzyme A dehydrogenase (*Mcad*) and carnitine palmitoyl transferase 1/2 (*Cpt1* and *Cpt2*), are targeted by PPARα protein and have crucial roles in fatty acid catabolism [9,10]. Under the insulin stimulation, sterol regulatory element-binding protein-1c (SREBP-1c), a major transcriptional regulator of fatty acid and triglyceride synthesis in the liver, will translocate to the nucleus and upregulate all genes in the fatty acid biosynthetic pathway, such as acetyl-CoA carboxylase (*Acc*), ATP-citrate lyase (*Acly*), fatty acid synthase (*Fasn*), and stearoyl-CoA desaturase 1 (*Scd1*) [11]. A previous study has reported that the increased level of nuclear SREBP-1c protein contributes to the elevated rates of hepatic fatty acid synthesis, leading to hepatic steatosis in diabetic mice [12].

AMP-activated protein kinase (AMPK) plays a central role in controlling lipid metabolism. The phosphorylation of ACC (at Ser79) by AMPK could inactivate ACC, which might ultimately decrease the level of malonyl-CoA and is beneficial for the recovery of CPT-1 activity and fatty acid oxidation [13,14]. The activity of SREBP-1c is also under the control of AMPK [15].

Apolipoprotein E (ApoE), primarily produced by the liver, works with other lipoproteins to mediate the lipid transport in the circulation [16]. ApoE knockout (KO) mice spontaneously develop hypercholesterolemia and atherosclerosis when fed a standard chow [17], while high-fat diet (HFD) can further accelerate the process [18]. Furthermore, previous studies have confirmed that ApoE KO mice fed a HFD could serve as a valuable NAFLD model [19,20].

Several lines of studies have shown that moderate hypoxia protocols had beneficial effects on metabolism, including reduced body weight, blood glucose, and cholesterol levels, and improved insulin sensitivity [21,22]. Hypoxia stimulates blood glucose disposal in rodents [23,24], isolated human muscle tissue [25], and type 2 diabetic patients [26]. High-altitude chronic hypoxia amends obesity-induced NAFLD in mice by regulating mitochondrial and AMPK signaling [27]. These findings imply that hypoxia exposure may be efficient for treating metabolic abnormalities. However, its possible effects on NAFLD have not been fully explored, and the conclusions regarding the potential relationship between hypoxia and NAFLD are inconsistent [28,29]. In particular, the effects of hypoxia-inducible factor 1α (HIF-1α), the master regulator of several genes responsible in cellular adaptation to hypoxia [30], on regulating hepatic steatosis setup and progression remain to be elucidated.

In this study, after 12 weeks of feeding of a HFD in ApoE KO mice, we focused on the effects of hypoxia exposure on fatty acid metabolism in the liver of the mice, and whether it would be more effective when shifting a HFD to a normal diet in this special animal model. We hypothesized that the intervention of hypoxia exposure would present beneficial impacts on hepatic fatty acid metabolism and HFD would suppress the beneficial effect in ApoE KO fed HFD.

## 2. Materials and Methods

### 2.1. Animals

The protocol of this study was approved by the Animal Care and Use Committee of Beijing Sport University (2019100A). Male C57BL/6J ApoE KO mice (*n* = 24, aged 10 weeks) and male C57BL/6J wild type (WT) mice (*n* = 6, aged 10 weeks) were purchased from Charles River Development, Inc. (Beijing, China). All mice were housed indoors under a temperature of 22 ± 2 °C, humidity of 50–70%, and 12-h light/dark cycles.

**Figure 1:**
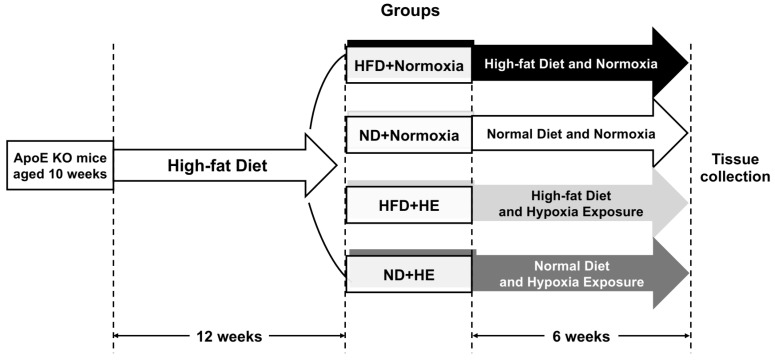
A schematic figure of the study protocol.

The ApoE KO mice were fed HFD, containing 21% (*w*/*w*) fat, 43% (*w*/*w*) carbohydrate without sucrose, and 1.5% (*w*/*w*) cholesterol, 4.554 kcal/g (Beijing Keao Xieli Feed Co., Ltd., Beijing, China) for 12 weeks. In the food protocol of for the first seven days, a HFD was gradually added into a normal diet until the feeding proportion was totally a HFD. After 12 weeks of the HFD feeding, all ApoE KO mice were forwarded into a six-week interventional experiment and randomly allocated to two groups: normoxia and hypoxia exposure (HE); then they were further divided into HFD and normal diet (ND) groups, respectively (Figure 1). There were four groups: HFD + normoxia, ND + normoxia, HFD + HE, and ND + HE, with six mice in each group (one mouse in the ND + normoxia group was euthanized due to accidental death in the later of intervention). The HFD groups were fed a HFD continuously, while the ND groups were changed to a normal chow containing 4–5% (*w*/*w*) fat, 50–60% carbohydrate, and no added cholesterol and sucrose, 3.420 kcal/g (Beijing Huafukang Bioscience Co., Ltd., Beijing, China). WT mice were fed a ND for 18 weeks as a baseline control.

Mice had *ad libitum* access to food. The hypoxia environment was created by placing the mice in a normobaric and hypoxia chamber (210 cm long, 200 cm wide, and 200 cm high) with an oxygen concentration of 11.2% (at about the level of a simulated altitude of 4500 m) together with the cage [31]. The chamber was infused with hypoxic air through an air compressor and a nitrogen synthesizing machine, which could reduce the oxygen concentration in the chamber to 11.2%. The oxygen concentration in the chamber was monitored throughout the experimental period with an oxygen sensor. The hypoxia treatments were carried out in the morning, one hour per time, three times per week, for six weeks. At the same time, the mice in the normoxia groups were placed in a normobaric and normoxic chamber together with the cage, and only the oxygen concentration was different between hypoxia and normoxia treatments.

To avoid acute effects of the last hypoxia treatment session, the mice were placed in normoxia at least 48 h prior to tissue collection. The mice were fasted overnight and were anesthetized (50 mg pentobarbital sodium/kg body weight) and the blood samples were collected by the percutaneous cardiac puncture. The liver samples from the left lateral lobe were removed, cleaned, and quick-frozen in liquid nitrogen, and then stored at −80 °C.

### 2.2. Body Weight, Food and Energy Intake

The mice were weighed weekly using an analytical scale. Any remaining food was replaced, and food intake was measured daily. The energy intake was calculated by energy content per gram of food × food intake.

### 2.3. Plasma Lipid Profiles

Plasma triglycerides (TG), total cholesterol (TC), low-density lipoprotein cholesterol (LDL-C), and high-density lipoprotein cholesterol (HDL-C) were measured following the methods and assay kits of our previous study [32]. Plasma non-esterified fatty acids (NEFA) were determined using a commercially available kit from Solarbio (BC0595, Beijing, China). Changes in absorbance were determined with Bio Tek Synergy H1 (Bio Tek Instruments, Inc., Winooski, VT, USA) at 550 nm.

### 2.4. Hepatic Lipid Contents, Plasma ALT and AST Levels

The supernatant of tissue homogenate was used to determine TG, TC, and NEFA in hepatic tissue by enzymatic methods according to the commercial kits (A111-1-1; A110-1-1; A042-2-1, respectively. Nanjing Jiancheng Bioengineering Institute, Nanjing, China). Plasma levels of alanine aminotransferase (ALT) and aspartate aminotransferase (AST) were measured using commercially available detection kits (C009-2-1 and C010-2-1, respectively. Nanjing Jiancheng Bioengineering Institute, Nanjing, China). Changes in absorbance were determined with Bio Tek Synergy H1 (Bio Tek Instruments, Inc., Winooski, VT, USA) at 510, 510, 546, 510, and 510 nm, respectively.

### 2.5. Real-Time Quantitative PCR Analysis

Total RNA was isolated from about 50 mg of liver tissue, reverse-transcribed to cDNA and performed real-time quantitative PCR analysis as previously described [33]. *Mcad* (QT00111244) and *18S ribosomal RNA* (*Rn18s*; QT02448075) commercial primers from Qiagen (Hilden, Germany) were used. The primer sequences of *Cpt1a*, *Cpt2*, *Acly*, *Acc1*, *Fasn*, *Scd1*, and *Srebp-1c* were listed in Table 1 and these primers were synthesized by Invitrogen Trading Co., Ltd. (Shanghai, China). The difference in expression between control and experimental samples was calculated using the 2^−ΔΔCt^ method, as described previously [34].

### 2.6. Western Blotting

According to the methods of our previous study, total proteins were isolated from 50 mg of liver tissue and western blotting was performed. The blots were probed using the following antibodies: HIF-1α (sc-10790, 1:1000), PPARα (sc-9000, 1:1000), SREBP-1 (2A4) (sc-13551, 1:500), DEC1 (s-8) (sc-101023, 1:1000), AMPKα1/2 (sc-74461, 1:1000), Thr172-p-AMPKα1/2 (sc-33524, 1:1000), and β-actin (sc-477778, 1:1000); the above-mentioned antibodies were all from Santa Cruz Biotechnology, Dallas, TX, USA. ACC (#3662, Cell Signaling Technology, Inc., Danvers, MA, USA) and Ser79-p-ACC (#3661; Cell Signaling Technology, Inc., Danvers, MA, USA). The density of protein bands was analyzed using Bio-Rad imaging software (Bio-Rad Laboratories, Hercules, CA, USA). The individual values were originally expressed as a ratio of a standard (β-actin content) and then expressed as a fold change of the control group value.

### 2.7. Activities of FASN and ACC

The activities of FASN and ACC in the liver tissue were determined using commercially available kits from Gene Lab (Beijing, China) and Solarbio (BC0415, Beijing, China), respectively. FASN catalyzed malonyl coenzyme A, acetyl coenzyme A, and NADPH to produce long chain fatty acids and NADP^+^. NADPH had a characteristic absorption peak at 340 nm. The activity of FASN was calculated by measuring the decreasing rate of absorbance at 340 nm (Bio Tek Synergy H1, Bio Tek Instruments, Inc., Winooski, VT, USA). ACC catalyzed acetyl CoA, NaHCO_3_, and ATP in the production of malonyl CoA, ADP, and inorganic phosphorus. Molybdenum blue and phosphate generated substances with characteristic absorption peaks at 660 nm (Bio Tek Synergy H1, Bio Tek Instruments, Inc., Winooski, VT, USA). The increase of inorganic phosphorus was measured by the ammonium molybdate phosphorus determination method to reflect the ACC activity.

### 2.8. Statistical Analysis

All values are reported as means ± SD. Statistical calculations were performed using SPSS Statistical software V 19.0 (IBM Corp., Armonk, NY, USA). Data were analyzed using a two-way ANOVA (ND × HE), and simple effect analysis with the least significant difference (LSD) post hoc test was performed to identify significant mean differences between groups. Significance was set at *p* < 0.05.

## 3. Results

### 3.1. Changes in Plasma Lipid Profiles, ALT, AST Levels and Hepatic Lipid Profiles between WT and NAFLD Model

**Figure 2:**
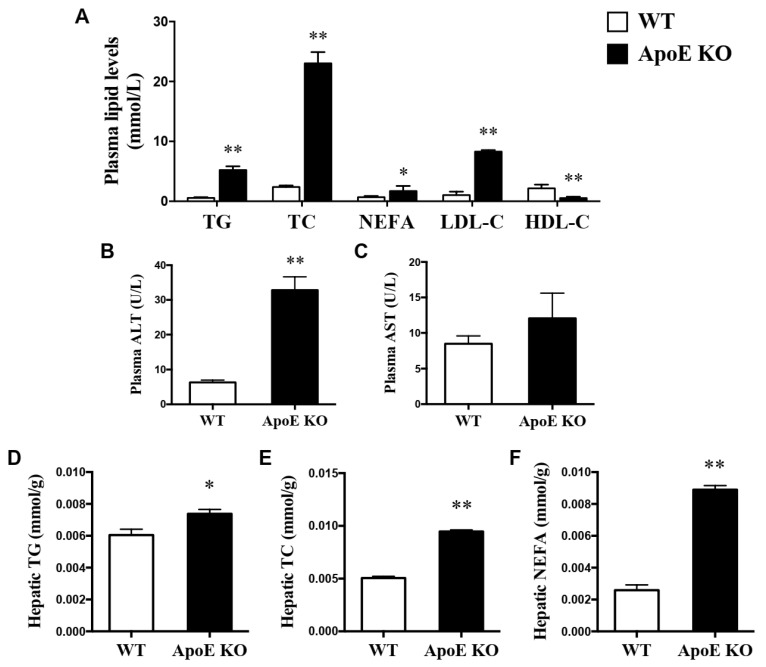
Changes in plasma lipid profiles (**A**), ALT (**B**), AST (**C**), and hepatic lipid profiles (**D**–**F**) between WT fed a ND and ApoE KO mice fed HFD. * *p* < 0.05, ** *p* < 0.01 vs. WT mice.

The ApoE KO mice fed a HFD had significantly higher levels (*p* < 0.05 or *p* < 0.01) of plasma TG, TC, NEFA, LDL-C, HDL-C, and ALT than those of WT mice fed a ND (Figure 2A,B). Furthermore, lower levels of hepatic TG, TC and NEFA were also found in WT mice than ApoE KO mice (*p* < 0.05 or *p* < 0.01) (Figure 2D–F).

### 3.2. Changes in Body Weight, Food and Energy Intake

**Figure 3:**
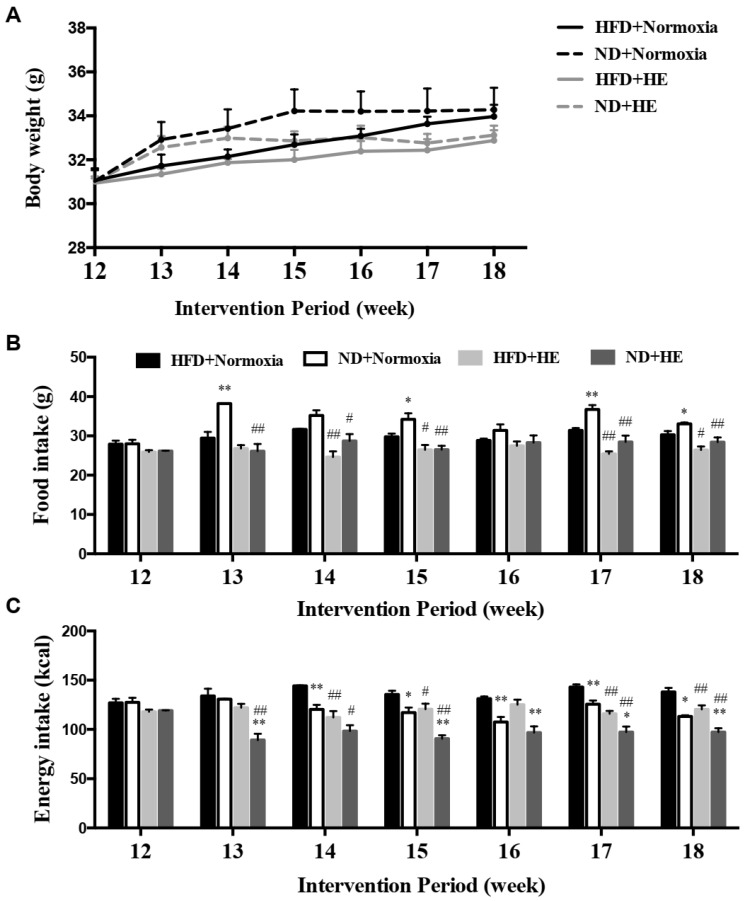
Changes in body weight (**A**), food (**B**), and energy (**C**) intake. * *p* < 0.05, ** *p* < 0.01 vs. HFD groups; # *p* < 0.05, ## *p* < 0.01 vs. normoxia groups.

There were no significant differences in body weight between the HFD and ND groups with or without HE during the six-week interventions (from the end of week 12 to the end of week 18) (Figure 3A). However, HE strongly decreased food and energy intake in the HFD + HE group compared with those of the HFD + normoxia group at 14, 15, 17, and 18 weeks of the intervention period, and also significantly reduced food and energy intake levels in the ND + HE group compared with those of the ND + normoxia group at 13, 14, 15, 17, and 18 weeks (Figure 3B,C). In addition, under normoxia, ND strongly increased food intake in the ND + normoxia group compared with those of the HFD + normoxia group at 13, 15, 17, and 18 weeks, but ND significantly decreased energy intake in the ND + normoxia group compared with that of the HFD + normoxia group at 14–18 weeks. Under hypoxia, ND also strongly decreased energy intake in the ND + HE group compared with those of the HFD + HE group at 13, and 15–18 weeks (Figure 3B,C).

### 3.3. Changes in Plasma Lipid Profiles, ALT, AST Levels and Hepatic Lipid Profiles

**Figure 4:**
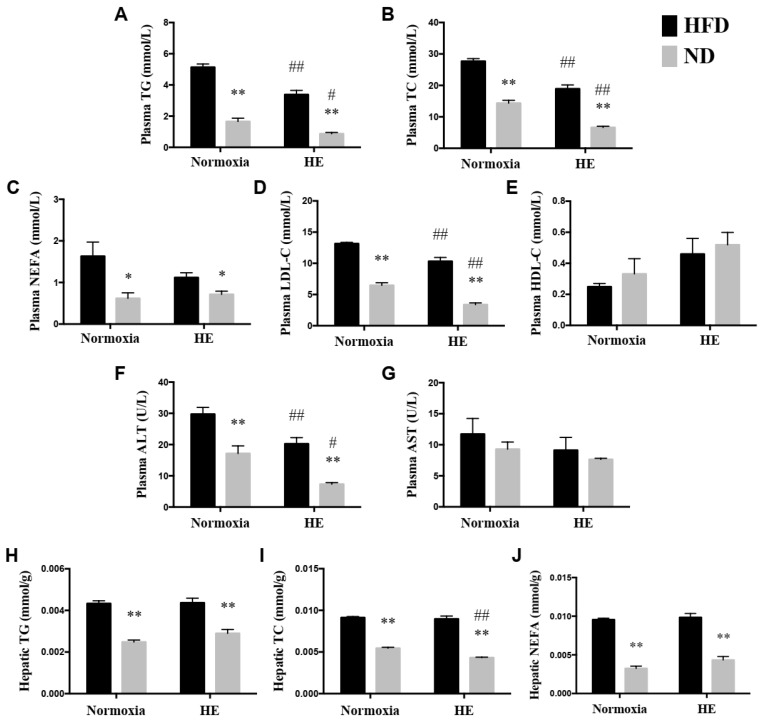
Changes in plasma lipid profiles (**A**–**E**), ALT (**F**), AST (**G**), and hepatic lipid profiles (**H**–**J**). * *p* < 0.05, ** *p* < 0.01 vs. HFD groups; # *p* < 0.05, ## *p* < 0.01 vs. normoxia groups.

HE strongly decreased plasma TG, TC, LDL-C, and ALT levels in the HFD + HE group compared with those of the HFD + normoxia group, and also significantly reduced plasma TG, TC, LDL-C, ALT, and hepatic TC levels in the ND + HE group compared with those of the ND + normoxia group. Under normoxia, ND strongly decreased plasma TG, TC, NEFA, LDL-C, ALT, and hepatic TG, TC, and NEFA levels in the ND + normoxia group compared with those of the HFD + normoxia group. Under hypoxia, ND also strongly decreased their levels in the ND + HE group compared with those of the HFD + HE group (Figure 4A–D,F,H–J).

### 3.4. Changes in Hepatic PPARα Protein Expression, mRNA Expression Levels of Genes Involved in Mitochondrial Fatty Acid Oxidation and Synthesis, and Activities of FASN and ACC

**Figure 5:**
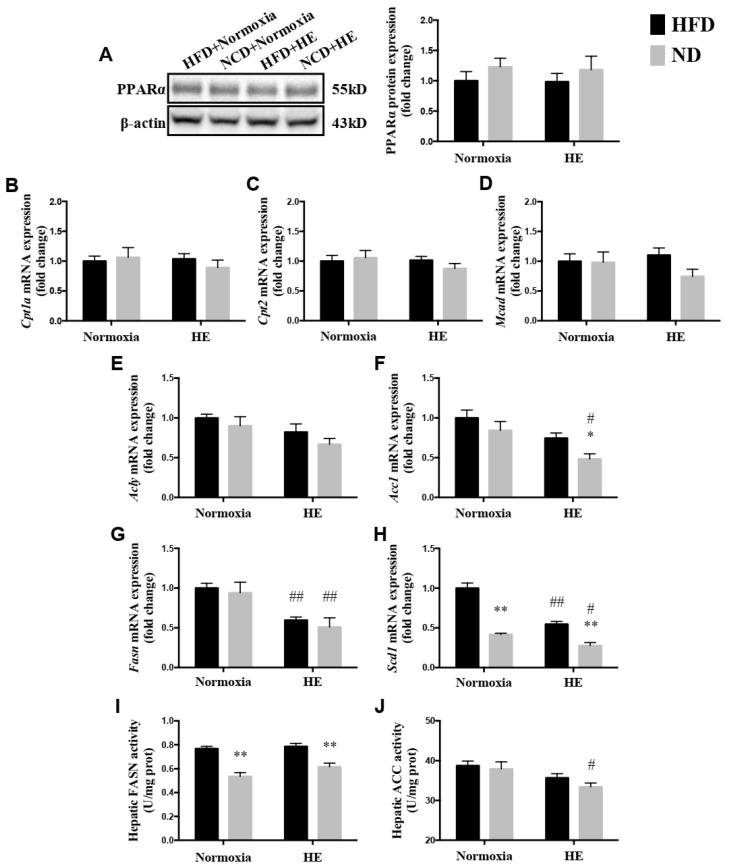
Changes in hepatic PPARα protein expression (**A**), mRNA expression levels of genes involved in mitochondrial fatty acid oxidation (**B**–**D**) and synthesis (**E**–**H**), and activities of FASN (**I**) and ACC (**J**). * *p* < 0.05, ** *p* < 0.01 vs. HFD groups; # *p* < 0.05, ## *p* < 0.01 vs. normoxia groups.

There were no significant differences in the protein expression of PPARα and mRNA expression levels of hepatic genes involved in mitochondrial fatty acid oxidation, including *Cpt1a*, *Cpt2*, and *Mcad*, between the HFD and ND groups with or without HE (Figure 5A–D). However, there were lower levels of the mRNA expression of *Fasn* and *Scd1* in the liver of the HFD + HE group than those of the HFD + normoxia group (Figure 5G,H). Additionally, the mRNA expression of *Acc1*, *Fasn*, *Scd1*, and ACC activity in the liver of the ND + HE group were lower than those of the ND + normoxia group (Figure 5F–J). In addition, ND strongly decreased hepatic mRNA expression of *Scd1* and FASN activity in the ND + normoxia group compared with those of the HFD + normoxia group, and ND also strongly decreased hepatic mRNA expression of *Acc1* and *Scd1*, and FASN activity in the ND + HE group compared with those of the HFD + HE group (Figure 5F,H,I).

### 3.5. Changes in Hepatic p-AMPKα(Thr172)/AMPKα and p-ACC(Ser79)/ACCα Ratios

**Figure 6:**
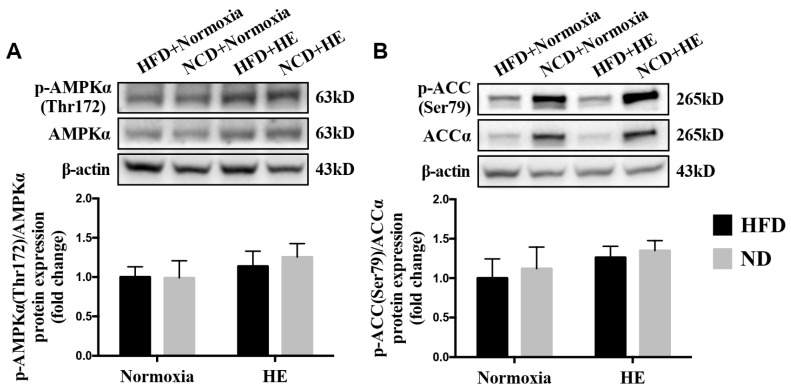
Changes in hepatic p-AMPKα(Thr172)/AMPKα (**A**) and p-ACC(Ser79)/ACCα (**B**) ratios.

To obtain more molecular evidence of the effects of HE on fatty acid metabolism, the protein expression of p-AMPKα(The172)/AMPKα, p-ACC(Ser79)/ACCα in the liver were measured. Immunoblotting revealed that there was an increasing trend in the hepatic p-AMPKα (The172)/AMPKα and p-ACC(Ser79)/ACCα ratios of the HFD + HE group or ND + HE group compared with the HFD + normoxia group or ND + normoxia group, respectively, but not significant between them (Figure 6A,B).

### 3.6. Changes in Protein Expression of HIF-1α, DEC1 and SREBP-1, and mRNA Expression of Srebp-1c

**Figure 7:**
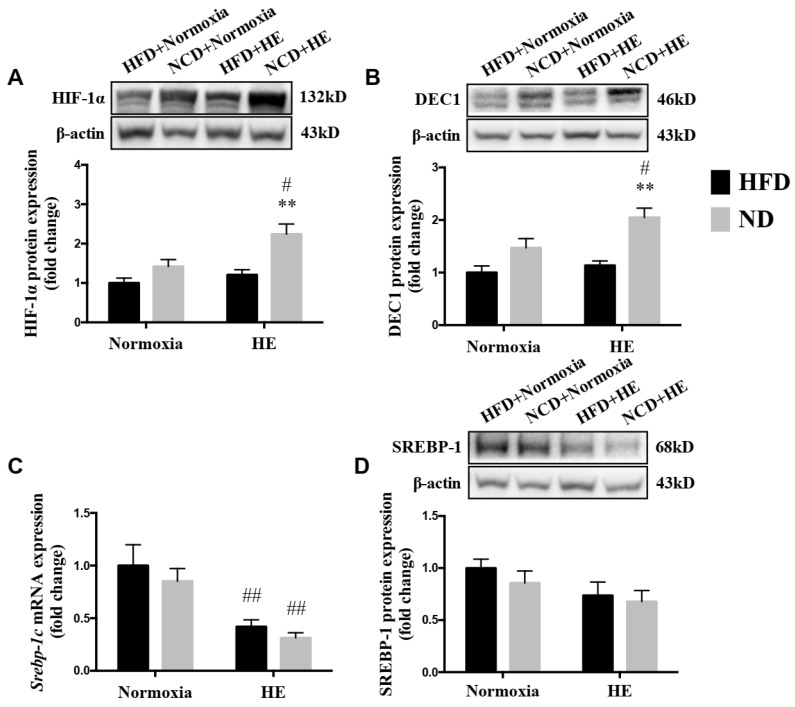
Changes in protein expression of HIF-1α (**A**), DEC1 (**B**), and SREBP-1 (**D**), and mRNA expression of *Srebp-1c* (**C**). ** *p* < 0.01 vs. HFD groups; # *p* < 0.05, ## *p* < 0.01 vs. normoxia groups.

There was a significantly lower level of the mRNA expression of *Srebp-1c* in the liver of HFD + HE mice, than that of the HFD + normoxia group (Figure 7C). Additionally, the protein expression of HIF-1α and DEC1 were higher, and the mRNA expression of *Srebp-1c* was lower in the liver of the ND + HE group than those of the ND + normoxia group (Figure 7A–C); the protein expression of HIF-1α and DEC1 in the liver of the ND + HE group were higher than those of the HFD + HE group (Figure 7A,B).

## 4. Discussion

The findings of the present study revealed that six weeks of hypoxia exposure improved the plasma lipid profile but did not affect hepatic TG and NEFA levels significantly in ApoE KO mice fed both a HFD and ND. Furthermore, the hypoxia exposure decreased the mRNA expression of *Fasn*, *Scd1*, and *Srebp-1c* significantly in the HFD + HE group compared with those in the HFD + normoxia group; while after replacing a HFD with ND feeding, the ND + normoxia group achieved more significant changes in the measured variables than those of the ND + HE group. In addition, the protein expression of HIF-1α was increased when hypoxia was performed in the ND group, but not in the HFD group. These results support the hypothesis of the present study at the genetic level, which is that the intervention of hypoxia exposure would present beneficial impacts on gene expression of hepatic fatty acid metabolism and HFD could suppress the beneficial effect caused by hypoxia treatment in ApoE KO mice, although at the macro level there were no significant changes in hepatic TG and NEFA contents following the hypoxia treatment. To the best of our knowledge, this study was the first investigation into the effects of hypoxia exposure on hepatic fatty acid metabolism in ApoE KO mice fed HFD.

Some studies have established that ApoE KO mice fed HFD could serve as a valuable NAFLD model [19,20]. Our results of blood lipid profiles, ALT, AST, and hepatic lipid contents also showed that ApoE deficient mice fed with HFD, compared with WT fed ND (Figure 2), lead to hepatic accumulation of free cholesterol and triacylglycerol and liver injury. This might suggest that ApoE KO mice could not correctly down-regulate dietary cholesterol absorption or stimulate biliary excretion when fed with a high-cholesterol diet [35].

The association between hypoxia and lipid homeostasis has already been speculated, however, the currently available data were inconsistent. Some studies have shown a lower prevalence of obesity in adult individuals living at moderate or high altitudes [36], and hypoxia could improve glucose-lipid metabolism disorders [24,27]. It is also known that high hypoxia may accelerate the progression of various diseases, such as cardiovascular disease, cancer, inflammatory diseases, and liver disease [37,38,39,40] and upregulate the gene expression involved in lipogenesis, lipid uptake, and lipid droplet formation [41,42]; while moderate altitude has the opposite influence since it reduces the prevalence and mortality of various diseases, such as cancer [43,44]. Previous studies have demonstrated that hypoxia exposure is associated with a loss of appetite and reducing food/energy intake [45,46]. In the present study, after six weeks of hypoxia exposure, ApoE KO mice fed both a HFD and ND significantly decreased their food and energy intake, and plasma TG, TC, LDL-C concentrations (Figure 3 and Figure 4). The suppressed food and energy intake by hypoxia could play a role in regulating these blood lipid profiles. Furthermore, the effects of hypoxia as a means of intervention on NAFLD development have not been sufficiently investigated and the precise mechanisms are not clear. Following six weeks of the hypoxia treatment, even though there were significant decreases in the mRNA expression of *Fasn* and *Scd1* in ApoE KO mice fed HFD and significant changes in more variables in ApoE KO mice fed ND (Figure 5), hypoxia exposure did not change hepatic TG and NEFA levels significantly in ApoE KO mice fed both a HFD and ND. We speculated that the insufficient dose and/or duration of hypoxia exposure would have an impact on its effects. In addition, we found that there were no significant changes in hepatic p-AMPKα(Thr172)/AMPKα and p-ACC(Ser79)/ACCα ratios, no matter whether in the intervention of hypoxia exposure or shifting a HFD to a ND, and hepatic ACC activity was decreased remarkably in the ND + HE group compared with that in the ND + normoxia group (Figure 6). It demonstrates that the phosphorylation of ACC can be regulated by other factors in hypoxia besides AMPK activation, such as the phosphorylation of ACC by protein kinase A (PKA) at Ser1200 which can also inhibit the enzymatic activity of ACC [14]. It is worth noting that there was a marked increase in the protein expression of ACC and p-ACC(Ser79) in the ND groups compared with that in the HFD groups, and their changes followed a similar trend during the trial. Further studies are required to elucidate the changes and their molecular mechanisms.

HIF-1 is known as the key oxygen-sensitive transcription factor, regulating most of the homeostatic responses to hypoxia. Recent studies have reported physiological and pathological effects of HIF-1 levels on the fatty liver. For example, HIF-1α may inhibit lipid accumulation by suppressing the SREBP-1c-dependent lipogenic pathway in the alcoholic fatty liver [47]. The findings were in line with previous reports of cell experiments, where HIF-1α upregulated the expression of differentiated embryo-chondrocyte expressed gene 1 (DEC1), a circadian helix-loophelix (HLH) transcription factor, which reduced the expression of SREBP-1c and its downstream lipogenic genes [47]. It has been consistently demonstrated that the ablation of HIF-1β promoted lipogenic gene expression (*Scd1, Fasn*) in the liver, which suggests that HIF-1 prevents lipid synthesis [48]. Protective effects of HIF-1 activation against fatty liver disease are further supported by a report in which HIF-1 promotes mitochondrial β-oxidation and prevents lipid peroxidation by regulating mitochondrial biogenesis in the liver of HFD-fed animals [49]. Collectively, these results indicate that HIF-1 serves as a protective factor against the development of fatty liver. However, the activation of HIF under hypoxia potentially leads to metabolic effects that are likely dose dependent, i.e., with modest hypoxia the results are beneficial; conversely, adverse effects will be shown if the exposure is extreme [47,50]. This is also supported by human studies reporting a deleterious metabolic effect of high altitudes vs. of beneficial influence of moderate altitude. In our study, the protein expression of HIF-1α was increased only when hypoxia was performed in the ND group, but not in the HFD group. ApoE KO mice fed HFD might be less sensitive to hypoxic stimulation, thus there was no difference in the expression of HIF-1 protein between the HFD + normoxia and HFD + HE groups (Figure 7). This question needs to be investigated in future studies. In addition, our data clearly indicated that the combined ND + HE treatment, compared with ND + normoxia or HFD + HE, enhanced the hepatic protein expression of HIF-1α and DEC1, reduced the mRNA expression of *Srebp-1c* and its target genes involved in fatty acid synthesis including *Acc1*, *Fasn*, and *Scd1*, as well as ACC activity (Figure 6 and Figure 7), which seemed to overwhelm lipid degradation caused by the induction of PPARα in ApoE KO mice.

Dietary intake of a HFD is a risk factor for the development of NAFLD [51,52]. Hepatocytes will accumulate fat when the cellular fatty acids input exceeds the fatty acid output in the case of a HFD, because the hepatic lipid levels are regulated by the interaction between liver absorption, synthesis, oxidation, and the export of lipids [53,54]. The plasma free fatty acids (FFAs) originating from the lipolysis of adipocytes and dietary fat are the primary source of hepatic lipid deposition in NAFLD [55]. Feeding HFD may lead to insulin resistance which would increase the rate of lipolysis in adipose tissue, resulting in elevated serum FFAs. This condition may accelerate the fat accumulation in the liver and finally reach a pathological state. In the present study, by changing a HFD to a ND under both hypoxia and normoxia conditions, there were significant decreases in plasma TG, TC, LDL-C, ALT, and hepatic TG, TC, and NEFA levels. Furthermore, the blood NEFA showed a similar trend as liver NEFA in the ND groups. The effects of a ND on the lower exogenous NEFA from adipose lipolysis could account for the lower liver TG level.

*De novo* lipogenesis (DNL) is a process by which lipids are endogenously synthesized from dietary sources, such as carbohydrates. SREBP has three isoforms: SREBP-1a, SREBP-1c, and SREBP-2. The liver predominantly expresses the SREBP-1c isoform together with SREBP-2. SREBP-1c is mainly involved in *de novo* FA and TG synthesis by inducing the expression of lipogenic genes such as *Fasn, Acc*, and *Scd1*, whereas SREBP-2 controls cholesterol homeostasis [56]. On the other hand, mitochondrial fatty acid β-oxidation (FAO) is a major route of lipid consumption in which FFAs are esterified with CoA, transported into the mitochondria matrix, and oxidized to generate acetyl-CoA by β-oxidation [57]. As expected, compared with the HFD group, the ND groups had a marked decrease in the activity of FASN and a significant reduction in the mRNA expression of *Scd1*, which can convert saturated fatty acids to monounsaturated fatty acids. Meanwhile, the protein expression of hepatic p-AMPKα(Thr172)/AMPKα and PPARα, the mRNA expression of PPARα’s target genes, such as *Cpt1a*, *Cpt2*, and *Mcad*, were not significantly different between the HFD and ND groups. Altogether, the evidence indicated that the dietary intervention with a ND could regulate hepatic fatty acid metabolism by inhibiting lipid synthesis.

There are no reports about hypoxia exposure improving hepatic fatty acid metabolism in humans with NAFLD in the current literature. However, in animal studies, chronic intermittent hypobaric hypoxia has beneficial effects on the body of rats [58,59]. This treatment protects the liver against hepatic damage through the inhibition of endoplasmic reticulum stress in fructose-fed rats [58] and has anti-diabetes effects through ameliorating insulin resistance via the hepatic HIF-insulin signaling pathway in type-2 diabetic rats [59]. The results of our present study provided evidence that six weeks of hypoxia exposure improved the plasma lipid profile, increased the protein expression of HIF-1α in the liver, and significantly affected hepatic metabolic indices of fatty acid synthesis at the genetic level in the male ApoE KO mice. In addition, the dietary intake of HFD is a well-established risk factor for the development of NAFLD. In the present study, by changing a HFD to a ND, there were significant decreases in plasma TG, TC, LDL-C, ALT, and hepatic TG, TC, and NEFA levels. Taken together, our results implied a prelusive possibility of using hypoxia treatment plus a dietary intervention to treat or prevent NAFLD in humans. Research on humans is needed to test this hypothesis in the future.

The present study has some limitations. While focusing on the changes of fatty acid metabolism in the liver, we did not assess other variables of lipid metabolism, for example, cholesterol metabolism. Future study may expand on the findings of cholesterol metabolism by addressing the effects and its mechanism. In addition, the liver histological observations were not performed, such as Oil Red O staining of samples in the study. This information would add more comprehensive insights. Because female mice and male mice have different hormone levels and physical conditions, the present study only selected male mice for the experiment to avoid the sex differences. The current results are not applicable to female animals.

## 5. Conclusions

Six weeks of hypoxia exposure did not change hepatic TG and NEFA levels in male ApoE KO mice. However, at the genetic level, there were significant effects on hepatic metabolic indices of fatty acid synthesis, especially in the ND + HE group, while HFD might suppress the beneficial effect of hypoxia on hepatic lipid metabolism in male ApoE KO mice. Comparatively, the dietary intervention of shifting HFD to ND was more effective in reducing hepatic lipid accumulation than hypoxia intervention.

## Figures and Tables

**Table 1:** Description of primers used for quantitative real-time PCR.

Gene Name	Gene ID	Forward Primer	Reverse Primer
*Cpt1a*	12894	CTCCGCCTGAGCCATGAAG	CACCAGTGATGATGCCATTCT
*Cpt2*	12896	CAGCACAGCATCGTACCCA	TCCCAATGCCGTTCTCAAAAT
*Acc1*	107476	ATGGGCGGAATGGTCTCTTTC	TGGGGACCTTGTCTTCATCAT
*Acly*	104112	ACCCTTTCACTGGGGATCACA	GACAGGGATCAGGATTTCCTTG
*Fasn*	14104	GGAGGTGGTGATAGCCGGTAT	TGGGTAATCCATAGAGCCCAG
*Scd1*	20249	TTCTTGCGATACACTCTGGTGC	CGGGATTGAATGTTCTTGTCGT
*Srebp-1c*	20787	GTGAGCCTGACAAGCAATCA	GGTGCCTACAGAGCAAGAG

## Data Availability

The data used to support the findings of this study are available from the corresponding author upon request.
